# CFD Model of a Mechanical Oscillator Flowmeter

**DOI:** 10.3390/s23010116

**Published:** 2022-12-23

**Authors:** Maciej Szudarek, Mateusz Turkowski, Adam Piechna

**Affiliations:** 1 Institute of Metrology and Biomedical Engineering, Warsaw University of Technology, 02-525 Warszawa, Poland; 2 Institute of Automatic Control and Robotics, Warsaw University of Technology, 02-525 Warszawa, Poland

**Keywords:** computational fluid dynamics, flow metrology, overset mesh

## Abstract

Most of the studies on mechanical oscillator flowmeters were conducted in the ‘80s and ‘90s when computational fluid dynamics (CFD) was not a viable scientific tool in flow metrology. Still, many topics related to the application of mechanical oscillator flowmeters require further investigation. In the article, a numerical model of a mechanical oscillator flowmeter is developed with the commercial software ANSYS Fluent. The model is validated against experimental data obtained at a water calibration stand. The influence of the selected turbulence model, dynamic mesh method, as well as grid and time step size is studied. The model’s qualitative behavior is correct, allowing investigation into the flowmeter operation in detail. It can provide a base for the improvement of the flowmeter’s performance. Relative differences in the frequency of oscillations did not exceed 4% for a DN50 flowmeter in the flow rate range (2–40) m^3^/h.

## 1. Introduction

Mechanical oscillator flowmeters, which appeared on the market in the 1970s, are simple, robust and, to some extent, resistant to dirt and sediments. They were introduced onto the market in response to the industry’s need for durable, accurate meters that would cover a wider measurement range than orifice flowmeters. Like the vortex and fluidic flowmeters, they utilize naturally occurring oscillations whose frequency varies with the flow velocity. Such a class of meters is often called oscillatory meters [1]. Unlike vortex-shedding flowmeters with a stationary bluff body, mechanical oscillator flowmeters have a bluff body which oscillates with the flow. Due to the large amplitude of oscillations, they are generally resistant to pipeline vibration and impacts, which are a constant problem in the case of vortex flowmeters [2,3]. In the case of a vortex flowmeter, a pressure signal generated by a vortex street is weak, it requires sophisticated methods of signal conditioning [4,5] and it results in increased susceptibility to pipe vibrations, e.g., those generated by pumps [6]. The strong, regular output signal in the whole flow rate range obtained using a vibrating magnet and a coil eliminates the necessity of the use of very sophisticated methods of signal conditioning.

In many applications, a mechanical oscillator flowmeter could be an economical alternative to other flowmeters; thus, in the authors’ opinion, it is worth making more effort to improve its performance. Although there is a general tendency to develop flow measurement technologies without moving parts (ultrasonic, magnetic, thermal), there is still a huge number of mechanical flowmeters manufactured and used, e.g., turbine, rotary, variable area, or positive displacement.

Early reports of a flowmeter based on a body oscillating with the flow date back to 1944 [7]. In this design, two angularly arranged rectangular blades were secured to a pivoted shaft, which extended centrally through a rectangular duct. Numerous improvements have been made concerning the shape of the oscillating body over the years. Ref. [8] presented prism-shaped and T-shaped oscillators. Besides patent descriptions, no experimental data on metrological or operational properties are available. Designs proposed in [9,10] were commercially manufactured in DN25, DN50 and DN80 variants. Eddy current proximity sensors detected the oscillations of the body. MPEs of 0.5% of the measured value were claimed for the measurement range of (4.8–48) m^3^/h for water, for the DN50 flowmeter [11]. The shortcoming of this design was that it significantly choked the flow in the neutral position of the oscillator. For that reason, an orifice was installed in a flowmeter body, which directed only part of the flow to the mechanical oscillator flowmeter. This introduced limitations in the case of the measurement of low flow rates and in the case of small pipe diameters. Refs. [12,13] studied a design of two wing-shaped bodies, one of which was stationary with the second being pivotally mounted. This device took advantage of a dynamic stall effect combined with a Venturi effect that drew the oscillator towards the fixed wing. It found applications in measuring large flow rates in rectangular ventilation ducts. Refs. [14,15] proposed the design of a vibrating ball for low flow rates. Ball-type designs were less popular, as they featured narrow gaps so that any foreign solid object could severely affect their performance, plus their error curves were highly nonlinear. Due to the simplicity of the geometry, their error curves could have been described with an analytical model [16]. Refs. [17,18,19,20] introduced a design which utilized a stream divider. The device was of simple construction, sufficient accuracy, and it was resistant to dirt and sediment to some extent. It was manufactured for DN15, DN25, DN50 and DN80 diameters by the Rota company. MPEs of 0.5% of the measured value were reported in the range of (4–40) m^3^/h for the measurement of water. The shortcoming was that the design required the proximity of walls to enhance and stabilize the oscillations. For that reason, the body of the flowmeter had a rectangular cross-section. The transition from a circular pipe shape to the rectangular cross-section and a wide oscillator were a source of significant pressure losses, which resulted in large values of pressure loss coefficient $\xi =2\Delta p/\left(\rho v^{2}\right)\approx 6.3$ for DN50, where Δ*p* is a pressure difference, *ρ* is fluid density and *v* mean flow velocity. Theoretical analysis of the geometry of the oscillator conducted by [21] showed that further optimization was possible. Although the analytical model was not very accurate, especially in terms of overestimated amplitude, it was quite effective for optimization. An optimized oscillator had higher energy of oscillations and no longer required wall proximity. Therefore, the oscillator could be narrower, and a flowmeter body with a circular cross-section could have been introduced. The pressure loss coefficient was reduced to 3.2 for the DN50 flowmeter. Additionally, the body of the flowmeter was five times shorter and lighter, which was especially important for larger nominal diameters.

There are also some studies regarding metrological and operational properties of mechanical oscillator flowmeters. In [22], the influence of density and temperature on the characteristics of the flowmeter was formulated. The lock-in phenomenon in the case of pulsatile flows was studied in [23,24]. The application of the flowmeter in a simple installation for multiphase flow measurement with the use of single-phase flowmeters was described in [25]. Baker’s handbook [26] suggested it would be interesting to see the further development of the practical use of such flowmeters.

Most of the studies were conducted in the ‘80s and ‘90s when computational fluid dynamics (CFD) was not a viable scientific tool in flow metrology. Still, many topics related to the application of mechanical oscillator flowmeters require further investigation. Detected lacunae include:

1. The determination of factors affecting the linearity of the error curve. Only preliminary studies on this issue exist.
2. Study on the influence of the energy of oscillations on the metrological and operational parameters of the flowmeter.
3. Study of the influence of inlet profile disturbances on the flowmeter’s performance. Existing studies require extension.
4. Study of the influence of pipe surface roughness on measurement characteristics. No such study has been conducted before.
5. Verification of equations describing the influence of fluid properties on the characteristics. Existing studies are limited to water and liquid sulfur.

The listed questions may be investigated with the aid of CFD modelling. As no CFD model of a mechanical oscillator flowmeter has been presented in the past, this article aims to assess the capabilities of CFD modelling and evaluate the predictive performance of various turbulence models and dynamic mesh methods.

In the paper, a CFD model of a mechanical oscillator flowmeter is formulated and validated against experimental data. First, introductory information about the principle of the operation of a mechanical oscillator flowmeter and its typical error curve is described. Then, the experimental stand and the studied oscillator shape are shown. The numerical model is defined and validated. Time step and grid sensitivity is studied, and different dynamic-mesh update methods and turbulence models are compared.

## 2. Materials and Methods

### 2.1. Principle of Operation of a Mechanical Oscillator Flowmeter

**Figure 1:**
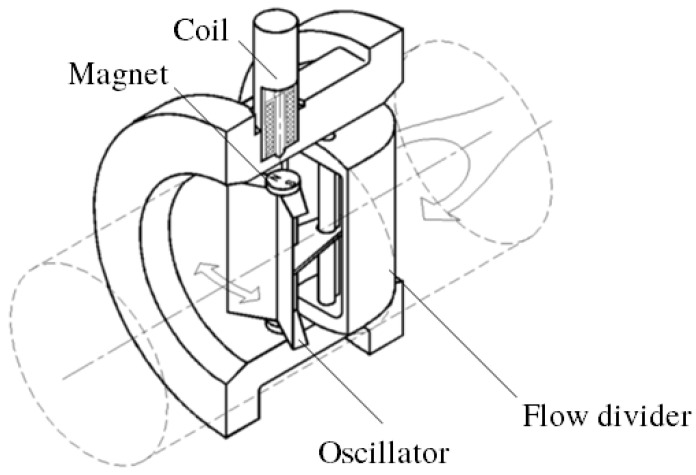
Main components of a mechanical oscillator flowmeter.

The main components of a mechanical oscillator flowmeter are shown in Figure 1. A flow divider splits the incoming fluid flowing in the pipe into two streams, guided past a pivotally mounted oscillator. The frequency of the oscillations is proportional to the volumetric flow rate and is measured, e.g., using a magnet attached to the oscillator and a coil.

**Figure 2:**
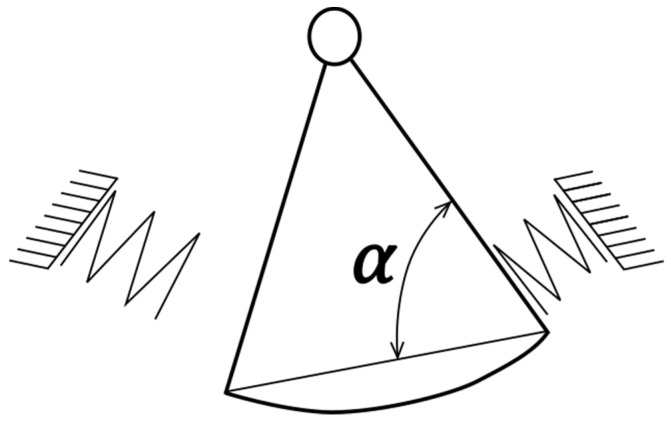
Analogy of the mechanical oscillator flowmeter to a compound pendulum; base angle *α* of the prismatic oscillator is marked.

The two streams can be regarded as fluidic springs, whose stiffness changes with the flow rate (Figure 2).

Following the analogy to a compound pendulum supported by springs, the angular frequency of the oscillator *ω* can be expressed with the equation:

(1) $$\omega =\sqrt{\frac{C_{\varphi }}{I}}$$

where $C_{\varphi }$ is the angular spring stiffness and *I* is the moment of inertia of the oscillator. The formula describing angular stiffness of “fluidic springs” was developed in [18] for the prismatic shape of the oscillator from the balance of forces, under the assumptions that the flow in the channel of the stream divider is uniform, and the flow is incompressible, two-dimensional, inviscid and stationary. In that case, the relationship between the oscillation frequency *f* and the volumetric flow rate *q_v_* takes the form:

(2) $$f=\frac{l_{0}q_{v}}{2\pi A}\sqrt{\frac{\rho h\sin2\alpha }{2I}}$$

where *l*_0_ is the level arm of the fluid force impinging on the oscillator, *A* = *hx* is the area of the flow cross-section that impinges on the oscillator, *h* is the height of the oscillator, *x* is the width of the oscillator immersed in the fluid stream, *ρ* is the fluid density, *α* is the angle marked in Figure 2 and *I* is the moment of inertia of the oscillator.

The resultant relationship between oscillation frequency and volumetric flow rate is linear. Such simplified reasoning concerns the case of an undamped oscillator and does not explain why the flowmeter operates in a limit cycle. Due to its numerous assumptions, neither does it allow the predicting of the calibration constant accurately. Therefore, calibration is required to determine the relationship between the flow rate and the frequency of oscillations. As shown in Equation (2), the frequency of oscillations depends on the density of the measured fluid. Similar to differential pressure flowmeter types [27,28,29,30,31], without knowing the fluid density no measurement can be carried out. This contrasts with vortex or ultrasonic flowmeters.

**Figure 3:**
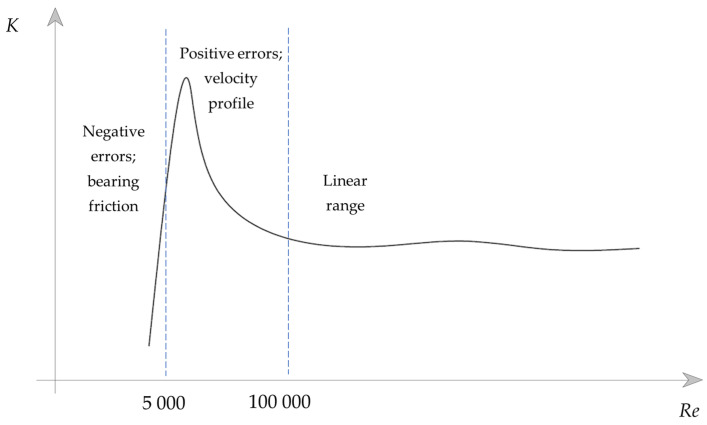
Typical calibration curve for a mechanical oscillator flowmeter in function of the Reynolds number.

Calibration constant *K* is the number of oscillations per volume of measured fluid, in pulses/m^3^. The Reynolds number is defined as:

(3) $$Re=\frac{\rho vD}{\mu }$$

where *ρ* is fluid density, *v* is the average velocity in the pipe upstream of the flowmeter, *D* is the internal pipe diameter, and *μ* is dynamic viscosity. A typical calibration curve of a mechanical oscillator flowmeter in the function of a Reynolds number is shown in Figure 3. Three regions are marked. At a certain threshold flow rate, the oscillator starts operating. Starting from the low-Reynolds-number region, initially, the bearing friction forces dominate, which is seen as a drop in the first part of the error curve. Below Re ≅ 1·10^5^, the velocity profile is sharpened, and it has more kinetic energy than a flat profile and, hence, a higher frequency of oscillations. It is suspected that this is the reason for the hump in the second zone [21]. Finally, the meter calibration coefficient remains relatively constant for higher Reynolds numbers.

### 2.2. Experimental Setup

Mechanical oscillator flowmeters come in many variants and this paper focuses on the design proposed in [32], as it reported promising metrological and operational properties and has been proven in industrial conditions. Maximum permissible errors do not exceed 0.5% for rangeability of 10:1 after characteristics linearization. This is worse than turbine flowmeters and better than orifice flowmeters.

Although the device has been manufactured for all the typical nominal diameters in the range of DN 15—DN 200, only a flowmeter of size DN 50 was selected for the study, as the available water flow facility fully covered its measurement range.

**Figure 4:**
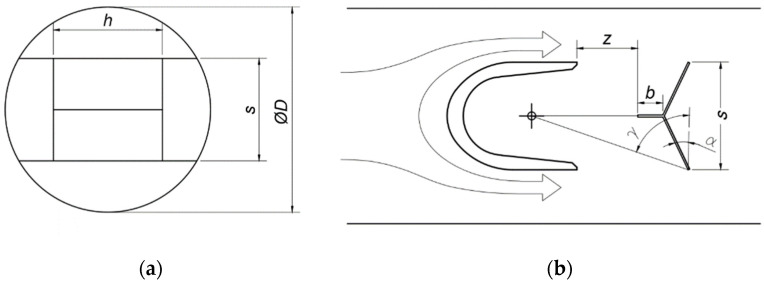
Parameters describing the oscillator’s shape: (**a**) rear view; (**b**) top view.

The geometry of the oscillator was recreated in CAD software, which allowed for calculating moments of inertia. Density was measured with a precise scale by comparison of the oscillator’s weight in air and water. Parameters describing the oscillator’s geometry are recalled in Figure 4 and their values are collected in Table 1.

In the previous studies [32], the frequency of oscillations was measured with a coil, which picked up the movement of a magnet attached to the oscillator. To measure the flow rate, this was sufficient. This study replaced the coil with a linear-output Hall sensor. It allowed capturing of not only frequency, but also the position of the oscillator in the function of time. This provided information on the amplitude of oscillations.

**Figure 5:**
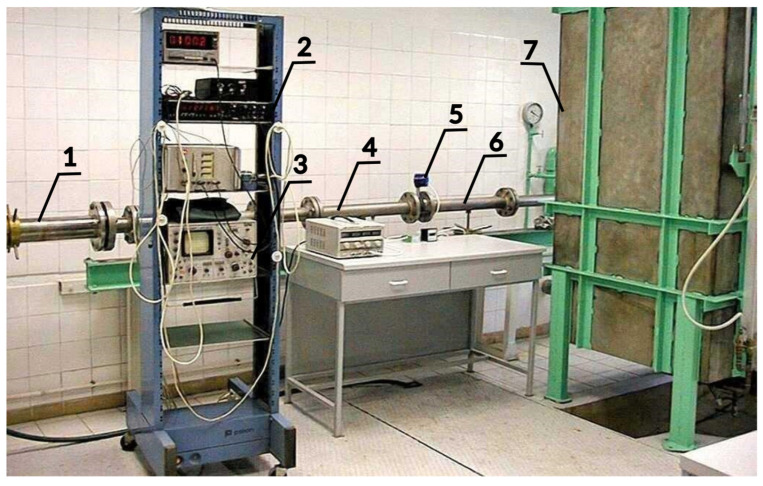
Water calibration stand with an installed mechanical oscillator flowmeter. 1—telescopic compensator, 2—pulse counter, 3—oscilloscope, 4—upstream pipe, 5—mechanical oscillator flowmeter, 6—downstream pipe, 7—measurement tank.

**Figure 6:**
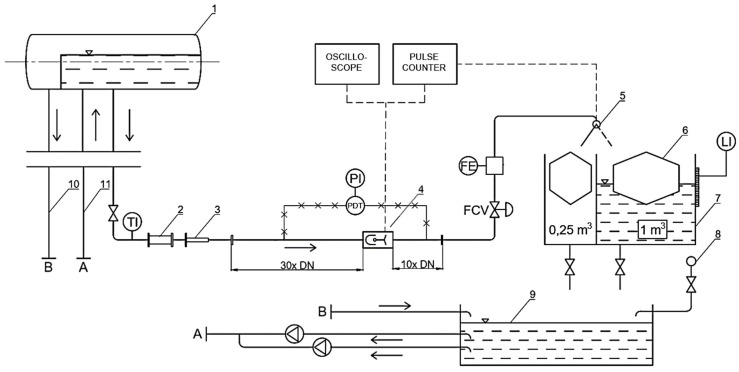
Water calibration stand diagram; 1—water supply reservoir, 2—in-line sight glass, 3—telescopic compensator, 4—mechanical oscillator flowmeter, 5—flow diverter, 6—fixed buoys which increase resolution of level measurement, 7—measurement tanks, 8—mains, 9—storage tank, 10—overflow pipe, 11—discharge line, TI—temperature indicator, PDT—pressure difference transmitter, PI—pressure indicator, FCV—flow control valve, FE—electromagnetic flowmeter, LI—level indicator. Dashed lines denote electrical connections. X-hatched lines denote pressure signal connections.

To determine the calibration constant of the flowmeter, a reference volume of water *V* was measured statically with tanks of 1 m^3^ and 0.25 m^3^ capacity. A gravity flow system was utilized to enhance flow stability and eliminate pulsations generated by pumps. An overview of the stand is presented in Figure 5 and a detailed diagram is shown in Figure 6.

As the mechanical oscillator flowmeter generates pulse output on the principle of its operation, it was possible to utilize a flying start and finish method. In this method, flow through the meter continues uninterrupted and a fast, electromagnetically driven diverter switches the flow between tanks. The pulse counter of the flowmeter was synchronized with the diverter. Therefore, volume *V* measured with tanks could be compared with the number of pulses generated by the oscillator. Precise time *t* measurement was also synchronized with the diverter, which enabled calculating the volumetric flow rate *q_v_*:

(4) $$q_{v}=\frac{V}{t}$$

The measurement of water temperature allowed for determining its density and calculating the mass flow rate. The water calibration stand allowed for study in the flow rate range (0.5–48) m^#^/h, with an expanded uncertainty of volume measurement *U_95_(V)* = 0.05%. Volumetric calibration constant *K* was calculated as the number of pulses *n* generated by the mechanical oscillator flowmeter divided by the reference volume of water *V*, which was measured with a volumetric tank:

(5) $$K=\frac{n}{V}$$

Combined uncertainty of the calibration constant *u(K)* is equal to:

(6) $$u\left(K\right)=\sqrt{{\left(\frac{\partial K}{\partial n}\cdot u\left(n\right)\right)}^{2}+{\left(\frac{\partial K}{\partial V}\cdot u\left(V\right)\right)}^{2}}$$

The uncertainty budget is presented in Table 2. Relative standard uncertainty of volumetric calibration constant *K* equals 0.025% and the expanded uncertainty for *k* = 2 equals 0.05%.

### 2.3. Numerical Model

The numerical model was developed with a commercial CFD code ANSYS Fluent 2019R2. Incompressible Reynolds-averaged Navier–Stokes equations (RANS) were solved using a finite-volume method. Pressure-based solver, implicit formulation and the Green–Gauss node-based option for the calculation of gradients were chosen for all the studied cases, except for overset mesh simulations, which supported only cell-based methods for gradient evaluation. A coupled scheme was applied for pressure–velocity coupling. Second-order spatial discretization for pressure, momentum, turbulent kinetic energy and turbulent dissipation rate was set.

Two- and three-dimensional numerical models of the mechanical oscillator were developed. Due to the circular pipe shape, only the three-dimensional model can predict the correct amplitude and frequency of the oscillations. However, the two-dimensional model allows to initially address some of the questions related to the design of the flowmeter: the role of the flow divider, the effect of fluid density, and the influence of the oscillator’s shape. There are some designs where the flowmeter body has a rectangular cross-section, e.g., the design by the Rota company. In such cases, a two-dimensional model would provide a better first approximation. Finally, there are existing analytical models derived for a planar case. With a two-dimensional numerical model, it will be possible to address the assumptions and limitations of these analytical models in future studies.

**Figure 7:**
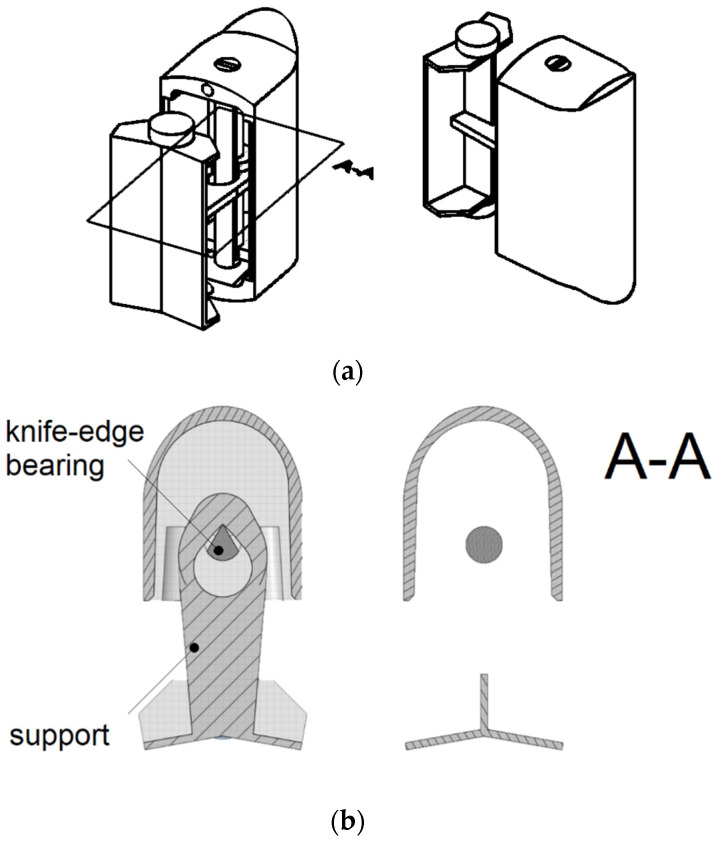
Geometry of the oscillator: (**a**) real geometry, isometric views, A–A symmetry plane is marked; (**b**) comparison of the real geometry in the A–A symmetry plane and the geometry of a 2D model.

The two-dimensional model corresponds to the cross-section of a real geometry in the symmetry plane. As depicted in Figure 7, minor changes in the geometry must have been introduced. The support has been removed. It would form an obstacle for incoming fluid that does not exist in real geometry. As such, it would bias the flow pattern and introduce damping in transient simulations. Moreover, the contact point between a stationary knife and a moving pan would pose a significant meshing difficulty. For that reason, the geometry of the bearing has been simplified to a cylinder which follows the rotation of the oscillator’s body.

To reduce the computational cost of a three-dimensional model, mirror symmetry was assumed and half of the geometry was used. For a single case a full model was run, and the oscillation frequency agreed with half of the model. The applied symmetry boundary condition assumes a zero flux (both convective and diffusive) in all quantities across a boundary. It was verified by comparison with a complete model that introducing symmetry does not noticeably affect the quantities of interest, such as the moment acting on the oscillator. Just like in the case of the two-dimensional model, the bearing geometry was reduced to a cylinder. Flow structures near the knife-edge bearing do not make a significant contribution to the moment; therefore, this simplification was justified. The rest of the geometry was left intact.

Inlet boundary conditions were in the form of fully developed profiles of velocity and variables required for turbulence modelling. Required profiles were obtained in separate simulations of an infinite length pipe, which was modelled by a periodic boundary condition.

**Figure 8:**
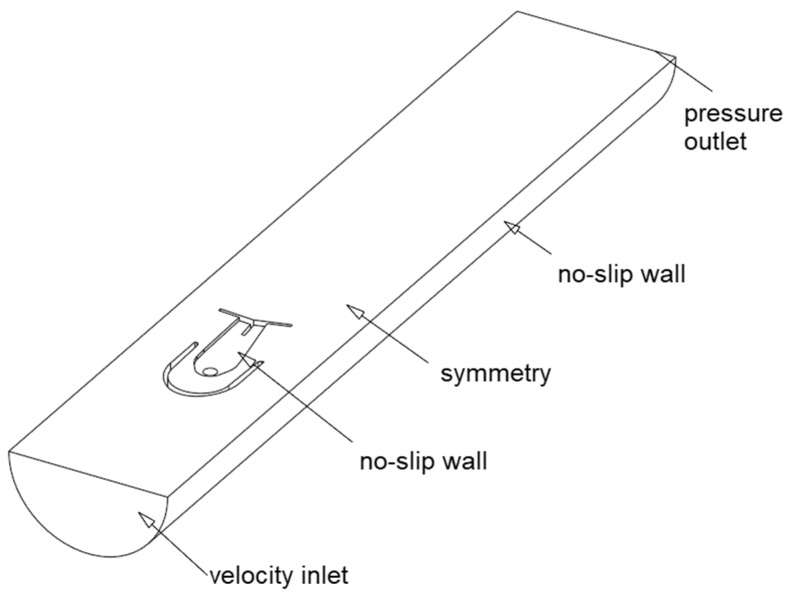
Boundary conditions for the three-dimensional case.

Figure 8 presents all the applied boundary conditions, that is, prescribed velocity and turbulence quantities at the inlet, zero-gauge pressure at the outlet, no-slip condition at walls and zero normal flux at the symmetry plane. Constant fluid density and viscosity of water were assumed.

The minimal size of the domain which does not affect the frequency and amplitude of oscillations was found. The influence of domain size downstream of the flowmeter on oscillator frequency is given in Table 3.

As shown, the outlet pipe length of three nominal diameters was sufficient. With the exact same procedure, a minimal required inlet pipe length was determined as 0.8*D*.

**Figure 9:**
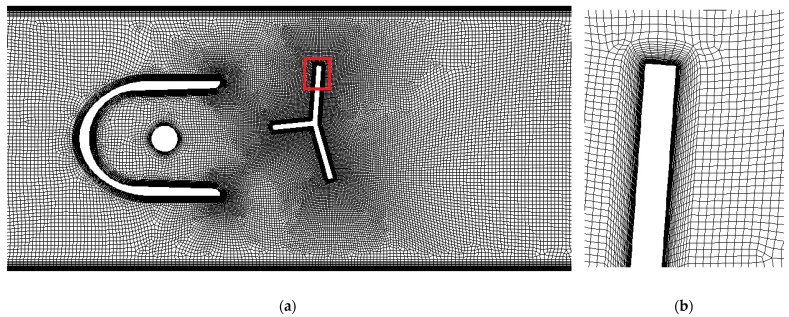
Two-dimensional mesh: (**a**) proximity of the oscillator, zoomed area marked in red; (**b**) boundary layers on the oscillator’s surface.

Both steady-state and transient simulations were run. An unstructured quadrilateral mesh was used for steady-state 2D cases with an immobilized oscillator, as shown in Figure 9.

**Figure 10:**
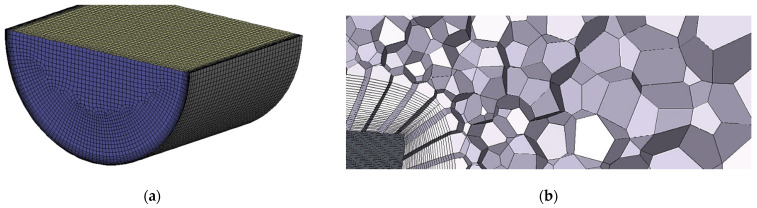

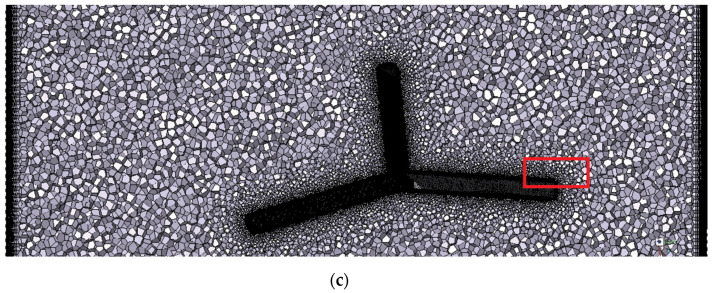
Three-dimensional mesh. (**a**) Inlet of the domain; (**b**) boundary layers on the oscillator’s surface; (**c**) the proximity of the oscillator, view of cross-section below the support. Zoomed area marked in red.

In the case of 3D simulations, inlet and outlet segments of the domain were meshed with swept hexahedral cells. Polyhedral cells were generated near the components of the flowmeter. Sizing functions were defined in the proximity of the oscillator and near the rear edge of the stream divider, as shown in Figure 10.

The quantity of interest was the moment acting on the oscillator. To compute the moment accurately, the viscous sublayer had to be resolved. The first layer thickness was adjusted depending on the boundary conditions to achieve dimensionless wall distance *y^+^* below 1 at the walls of the stream divider and oscillator. This meant that the first mesh element was placed in the viscous sublayer. The rest of the boundary layer mesh consisted of 15–30 prism elements with a growth rate not higher than 1.15.

For all the studied cases, normalized mesh quality parameters were monitored. Mesh skewness did not exceed 0.9 and the orthogonal quality was above 0.1, which means a very good quality of meshes.

In the case of transient simulations, the oscillator was no longer stationary. In each time step, the moment acting on the oscillator was used to compute the angular motion. Then, the computational mesh had to be adjusted to its new position, taking into account the moment of inertia of the oscillator. Various dynamic mesh methods were considered:

- Smoothing and remeshing—computational mesh stretches or shrinks in a spring-like manner. If the deformation causes a decrease in mesh quality below acceptable levels, local remeshing is activated. The main disadvantages are variable mesh quality and computational time.
- Layering—computational mesh collapses or is generated layer by layer. This method is the fastest and it is advantageous because of mesh quality, but the requirement of a hexahedral grid limits applications to simple geometries and motions (pure rotation, pure translation).
- Overset mesh [33]—multiple grids, related to background and moving components, are superimposed on each other. Overset interfaces connect fluid zones by interpolating cell data in the overlapping regions. The advantage is that a moving body mesh and a background mesh can be generated independently, with higher quality and fewer constraints than if the system was meshed all at once. However, the interpolation between grids is not conservative, so results should be examined carefully.

**Figure 11:**
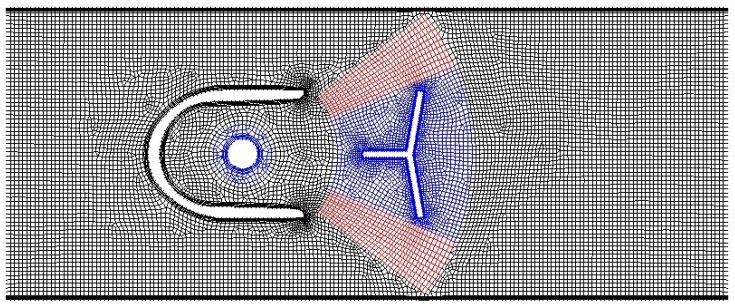
Two-dimensional mesh for the layering method: black—stationary zone; red—layering zone; blue—rigid zone.

For the 2D model, the geometry was simple enough to apply the layering method. The mesh was decomposed as shown in Figure 11. Cell zones in the proximity of the oscillator and the pan are rotating rigidly with them.

**Figure 12:**
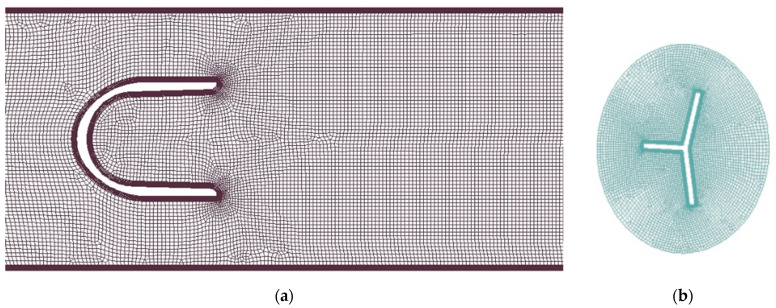

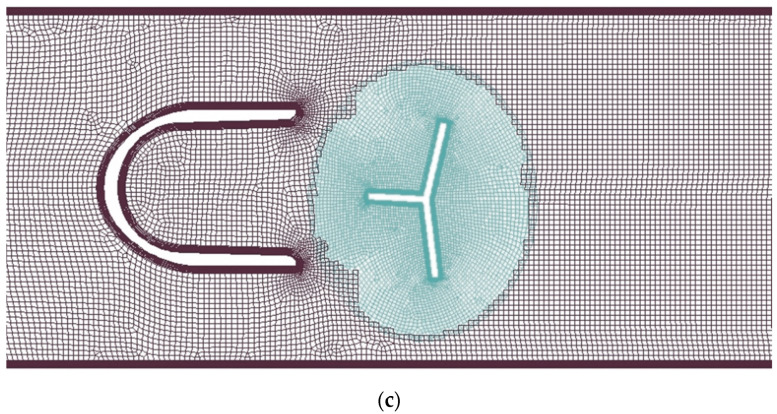
Two-dimensional mesh for the overset method: (**a**) background mesh; (**b**) component mesh, which moves as a rigid body with the oscillator; (**c**) superimposed grids, an overlapping interface between grids is visible.

The angular extent of layering zones limits the maximum angular position of the oscillator. It was not an issue in two dimensions, but in the case of a 3D model the walls in the extreme position of the oscillator were too close to each other to isolate the structured zones of the mesh while maintaining a reasonable boundary layer. Therefore, in the 3D case, the choice was between remeshing and overset mesh. Remeshing requires tetrahedral cell elements, which are usually less efficient than polyhedral; therefore, the overset method was chosen. To verify the overset approach, a comparison between overset and layering methods was made for 2D. The idea of the overset mesh method in this application is shown in Figure 12. After verifying that the overset method yields accurate results, all the dynamic mesh cases for the three-dimensional geometry were calculated using the overset approach.

The moment acting on the oscillator was the monitored value throughout the iterative calculation process. Iterative calculations were run until iterative convergence of moment was reached, which in the transient case usually took place after no more than 30 iterations per time step.

**Figure 13:**
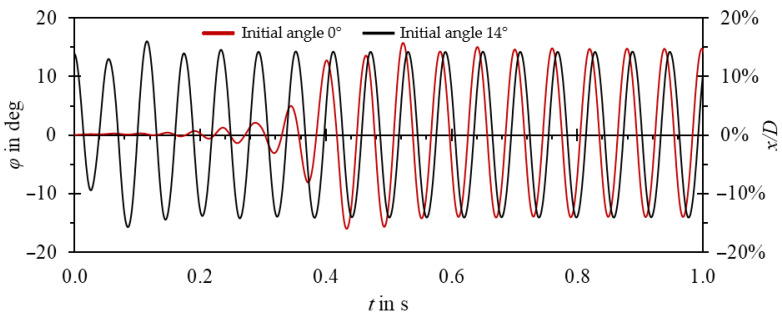
Oscillator position vs. time for different initial positions of the oscillator. Red line—starting from *φ* = 0°, black line—starting from *φ* = 14°.

As the oscillator operates in a limit cycle, the amplitude and frequency of oscillations do not depend on the initial position of the oscillator. Thus, the initial position in simulations could have been set close to the expected amplitude. As shown in Figure 13, this allowed reducing computation time. Periodic oscillations were achieved twice as fast in comparison to initialization with a neutral position of the oscillator.

Grid sensitivity was assessed in steady state both for 2D and 3D cases. Representative cell size *h* was defined as the local cell size in the proximity of the oscillator. Multiple gradually refined meshes with refinement factor *h_coarse/_h_fine_* = 1.3 and with the same topology were studied. The summary of the studied cases is given in Table 4.

## 3. Results and Discussion

**Figure 14:**
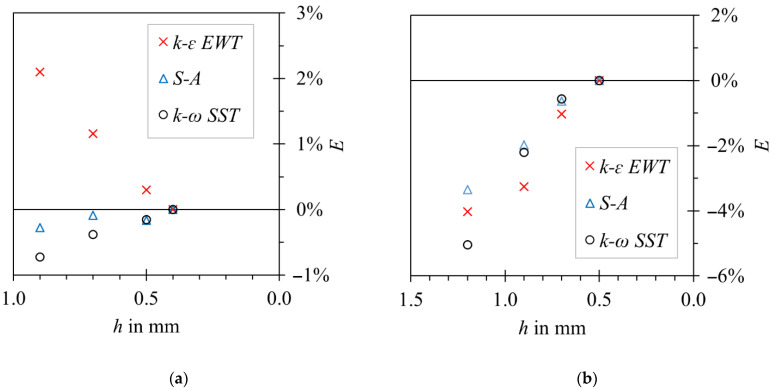
Steady-state grid independence test, relative differences between moment coefficient for given mesh size and the reference mesh. (**a**) Two-dimensional model; (**b**) three-dimensional model.

The results of the grid sensitivity test are shown in Figure 14. Differences between S-A, realizable *k-ε* and *k-ω* SST turbulence models were in the range of 1.5% in favor of the *k-ω* SST model. For that reason, this turbulence model was selected for the rest of the studied cases. Considering the number of simulations that had to be conducted, the G3 grid was selected for two-dimensional cases and the G2 grid was selected for three-dimensional cases, as a compromise between computational effort and solution accuracy. Well-defined flow separation lines can explain the low dependency on the applied turbulent model on the oscillator’s body.

There were several criteria to consider when selecting the time-step size. In the layering method, the time step should be such that the relative mesh motion is at most the length of the smallest cell at the dynamic mesh interface.

Additionally, the Courant–Friedrichs–Lewy condition describes the convergence condition of partial differential equations:

(7) $$CFL=\frac{v\Delta t}{\Delta x}\leq CFL_{MAX}$$

where *v_i_* is fluid velocity, Δ*t* is time step size and Δ*x* is cell size. Implicit solvers, which were used in the simulation, are not strictly limited by *CFL* and permit values of *CFL_MAX_* in the range of 200.

Finally, increasing the time step decreases solution accuracy. There is a question of how many time steps per oscillation period are required. Therefore, a time-step sensitivity study was performed.

The observed variables were mean oscillation frequency $\bar{f}$ and amplitude $\bar{A}$. Twenty stable periods of oscillations were captured for each case. A comparison between results obtained with different dynamic mesh methods for the G3 grid is given in Table 5.

In the case of the layering method, 4 × 10^−4^ s was the largest permissible time step, as for higher time steps the solution diverged. The overset mesh method allowed for larger time steps, but that would introduce an additional 2% error in oscillation frequency.

Below 4 × 10^−4^ s, the layering method was not sensitive to the time-step value and the results were comparable, regardless of the order of time discretization. The overset mesh method required a smaller time step to maintain accuracy.

**Figure 15:**
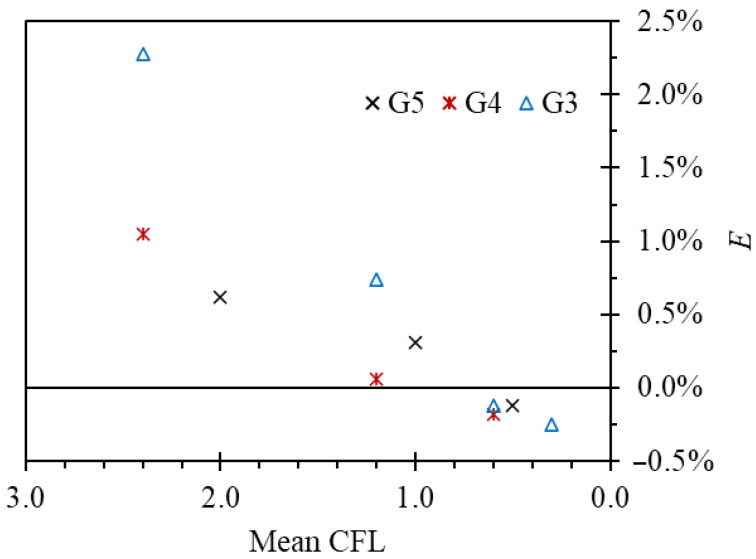
Influence of time-step size on the oscillation frequency for the overset mesh method in reference to results obtained with the layering method of the same time-step size and grid size. Series correspond to different grid sizes.

Further tests confirmed the requirement of the mean *CFL* number to be below 1 for the overset case (Figure 15).

**Figure 16:**
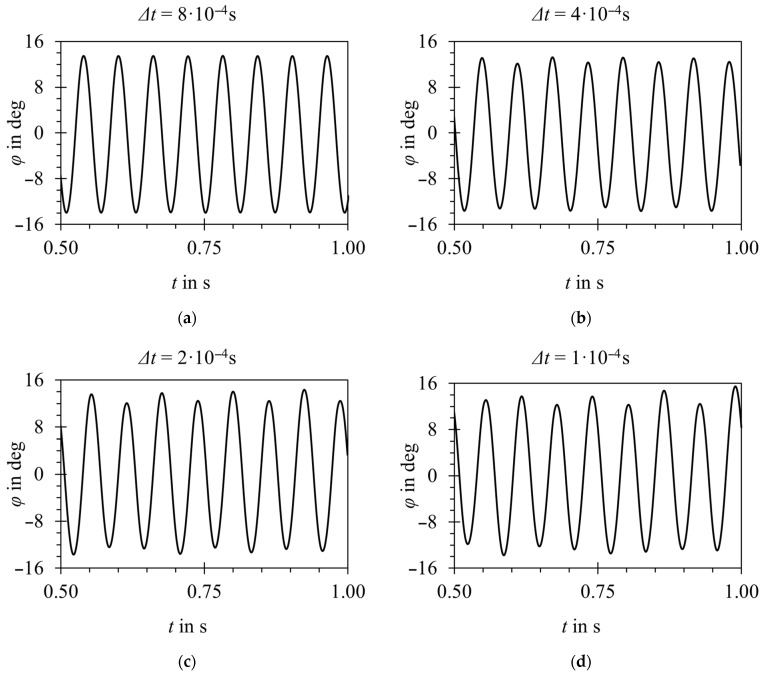
Oscillator position vs. time for the two-dimensional model, G3 grid, and various time-step sizes. (**a**) 8 × 10^−4^ s; (**b**) 4 × 10^−4^ s; (**c**) 2 × 10^−4^ s; (**d**) 1 × 10^−4^ s. Increased jitter with reducing time step is visible.

As another example of time-step sensitivity, it was observed that with increasing time step, parts of the velocity field are filtered out. As a result, amplitude and frequency jitter is damped with increasing time-step size, as illustrated in Figure 16 for the example of the two-dimensional model.

**Figure 17:**
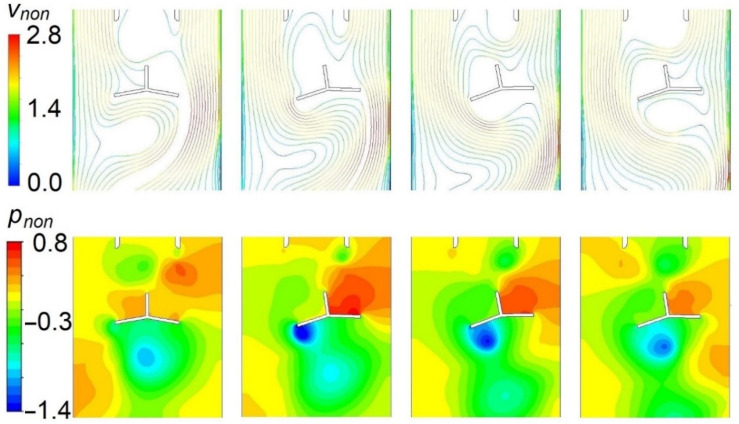
Two-dimensional model, a sequence of images over a quarter of an oscillation cycle. Top—pathlines colored by nondimensional velocity (normalized by inlet velocity). Bottom—contours of nondimensional pressure (normalized by dynamic pressure).

Figure 17 shows images over a quarter of an oscillation cycle. As the oscillator enters the stream, a vortex builds up on the side of the oscillator that is hidden behind the stream divider. It causes a local decrease in pressure and an increase in velocity. As the oscillator reaches the extreme angular position and starts to move back, the vortex detaches and is swept with the flow.

**Figure 18:**
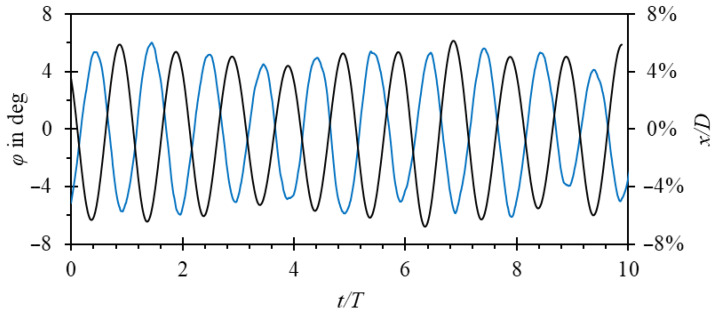
Position of the oscillator vs. time for *q_v_* = 40 m^3^/h. Black line—three-dimensional numerical model, blue line—experiments. Series are offset by 0.5*T* intentionally, for clarity reasons.

Amplitude jitter was observed in the experiment. As shown in Figure 18, a deviation from the true periodicity of oscillations is present in both amplitude and frequency. Providing that the time step and mesh is small enough, the numerical model captures that effect correctly. Amplitude jitter was also confirmed with visual observations by mounting the oscillator in an acrylic glass body. This constitutes significant progress compared with the analytical model developed in [32], where the amplitude of oscillations was overestimated about twice. It seems that this was due to the omission of the phenomena occurring downstream the oscillator in the analytical model.

**Figure 19:**
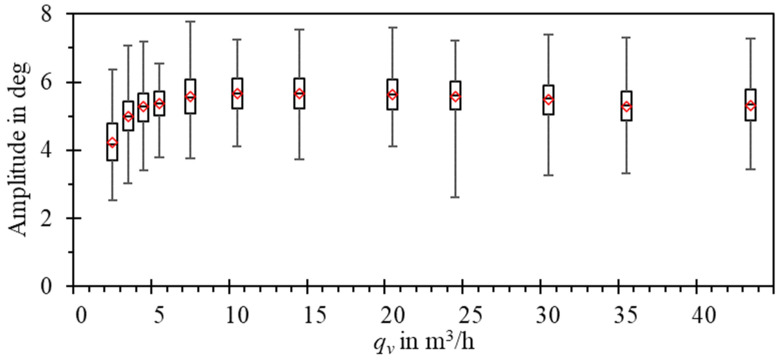
Box-whiskers plot of the amplitude of oscillations vs. flow rate, measured experimentally at the water calibration stand.

The experimental study showed that the mean amplitude of oscillations is constant for *Re* > 65,000 (ca. 8 m^3^/h in Figure 19) and drops from 5.5 degrees to ca. 4.2 degrees for lower values of the Reynolds number. Although the mean amplitude for a given flow rate remains constant, the spread of captured oscillation amplitudes reaches up to 3.5 degrees.

**Figure 20:**
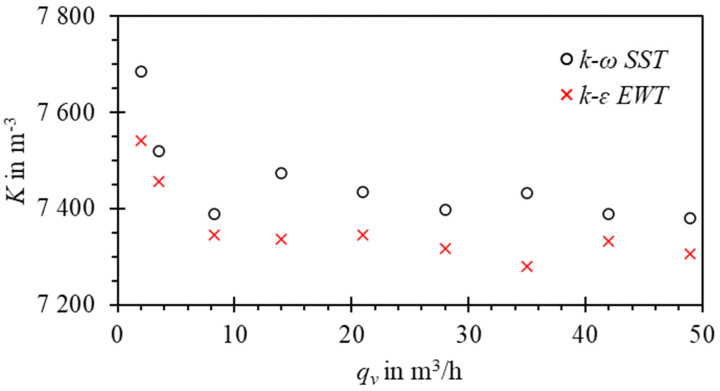
Error curve obtained with the two-dimensional model, overset method, G3 grid, and mean CFL < 1.

In the two-dimensional case, the predicted amplitudes were ca. 2 times larger than in the three-dimensional case. The 2D model could not capture the circular pipe shape and wall-proximity effects that limit the amplitude of oscillation in the real case. For that reason, it was not possible to relate 2D simulation results to the experiment. Nevertheless, the shape of the error curve $K\left(q_{v}\right)=\bar{f}/q_{v}$ was qualitatively correct, as shown in Figure 20. Increased values of calibration constant *K* for low Reynolds numbers are followed by a nearly constant value above *q_v_* = 10 m^3^/h. The differences between the results obtained with different turbulent models in 2D did not exceed 2.5%.

**Figure 21:**
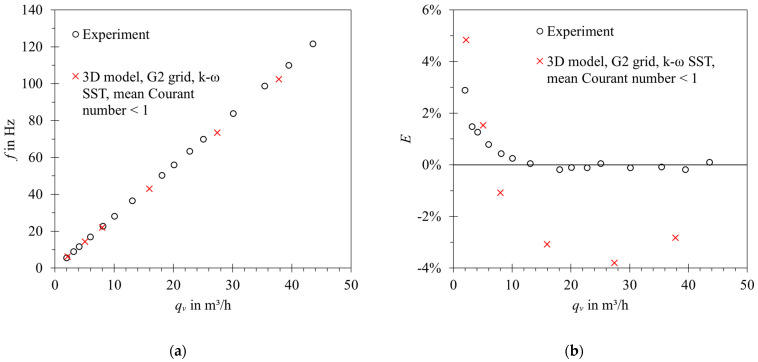
Validation of the three-dimensional model against experimental data of the flowmeter: (**a**) Frequency vs. volumetric flow rate; (**b**) error curves for *K* = 10,03 imp/dm^3^. Error bars denote experiment uncertainty for coverage factor *k* = 2.

In the 3D case, it was possible to relate the results to experimental values, as shown in Figure 21.

The relative differences between the model and experimental data did not exceed 4%. The differences were primarily attributed to mesh sensitivity and modelling errors, e.g., applied turbulence models. The agreement between the error curves’ shape for low Reynolds number can be further improved by introducing bearing friction to the numerical model. The results were considered satisfactory for further studies. The qualitative behavior of the model is correct, and it allows both studying the flowmeter operation in detail and performing parametric studies.

## 4. Conclusions

A CFD model of a DN50 mechanical oscillator flowmeter was developed and validated experimentally. Its sensitivity to grid and time-step size was assessed and two dynamic mesh methods, overset mesh and layering, were compared. Both dynamic mesh methods yielded similar results, given that the time step was low enough. Below mean CFL < 1, the time-step dependency on results was in the range of 0.5%. For higher time steps, errors up to 2.5% were observed. Amplitude and frequency jitter were captured, which was also observed in the experiment. Differences between studied RANS-based turbulence models were in the range 2.5%. Well-defined flow separation lines can explain the low dependency on the applied turbulent model on the oscillator’s body. In the case of the three-dimensional model, the relative differences in frequency of oscillations did not exceed 4% for a DN50 flowmeter in the flow rate range (2–40) m^3^/h.

The developed numerical model of the mechanical oscillator flowmeter has not yet been described in the literature and it represents significant progress compared to previously available analytical mathematical models. It opens up the possibility of the further optimization of the mechanical oscillator flowmeter. More information about installation requirements can be found with the numerical model. Means to improve the linearity of the flowmeter characteristics can be found. Correction factors for density and temperature should be further investigated with the use of experiments on different fluids and using CFD. The shape of the oscillator can be optimized concerning the energy of oscillations. Larger oscillation energy would provide a wider measurement range and smaller sensitivity to flow disturbances and measured fluid properties. The shape of the flow divider can also be modified, balancing the sensitivity of the flowmeter to upstream flow disturbances and introduced pressure losses.

## Figures and Tables

**Table 1:** Parameters of the studied oscillator.

Geometry							Mass Properties		
*D*	*s/D*	*α*	γ	*b/D*	*z/D*	*h/D*	Material	Mass *m*	Moment of Inertia *I_o_*
50 mm	43.3%	10°	70.1°	15%	20%	78%	austenitic steel	17,352 g	1.06 × 10^−5^ kg·m^2^

**Table 2:** Uncertainty budget for calibration using water.

Quantity	Unit	Value *x_i_*	*u(x_i_)*	Probability Distribution	Sensitivity Coefficient *c(x_i_)*	*c(x_i_)·u(x_i_)* in Pulse/dm^3^	% of Total
*n*	pulse	10,060	0.41	triangular	0.001	0.00041	3%
*V*	dm^3^	1000	0.25	normal	−0.01	−0.0025	97%
*K*	pulse/dm^3^	10,060	0.0025				100%

**Table 3:** Study of the required length of a straight outlet run.

Domain Size Downstream in Nominal Diameters	Shift in Oscillation Frequency Related to the Largest Domain
0.5	1.60%
1	−0.13%
2	0.06%
3	0.00%
6	reference

**Table 4:** Mesh independence study details.

Grid Number	Representative Cell Size in mm	Element Count 2D Model	Element Count 3D Model
G1	1.2	-	3.8·10^6^
G2	0.9	3.3·10^4^	5.9·10^6^
G3	0.7	4.7·10^4^	8.0·10^6^
G4	0.5	8.0·10^4^	1.1·10^7^
G5	0.4	1.2·10^5^	-

**Table 5:** Time-step sensitivity study for the two-dimensional case.

			Overset Mesh, 1st Order Time Discretization		Layering, 1st Order Time Discretization		Layering, 2nd Order Time Discretization	
Δ*t* in s	Time Steps per Oscillation Cycle	Mean CFL	$\bar{A}$ in deg	*f* in Hz	$\bar{A}$ in deg	*f* in Hz	$\bar{A}$ in deg	*f* in Hz
8 × 10^−4^	75	2.4	13.7	16.63	-	-	-	-
4 × 10^−4^	153	1.2	13.0	16.38	12.9	16.25	13.6	16.26
2 × 10^−4^	308	0.6	12.9	16.24	13.6	16.26	12.7	16.24
1 × 10^−4^	617	0.3	13.1	16.22	12.9	16.26	12.9	16.26

## Data Availability

Not applicable.
